# Krüppel-like Factor 5 contributes to pulmonary artery smooth muscle proliferation and resistance to apoptosis in human pulmonary arterial hypertension

**DOI:** 10.1186/1465-9921-12-128

**Published:** 2011-09-27

**Authors:** Audrey Courboulin, Véronique L Tremblay, Marjorie Barrier, Jolyane Meloche, Maria Helena Jacob, Mathilde Chapolard, Malik Bisserier, Roxane Paulin, Caroline Lambert, Steeve Provencher, Sébastien Bonnet

**Affiliations:** 1 Department of Medicine, Faculty of Medicine, Laval University, Quebec QC, Canada

**Keywords:** Pulmonary arterial hypertension, KLF5, STAT3, proliferation, apoptosis.

## Abstract

**Background**

Pulmonary arterial hypertension (PAH) is a vascular remodeling disease characterized by enhanced proliferation of pulmonary artery smooth muscle cell (PASMC) and suppressed apoptosis. This phenotype has been associated with the upregulation of the oncoprotein survivin promoting mitochondrial membrane potential hyperpolarization (decreasing apoptosis) and the upregulation of growth factor and cytokines like PDGF, IL-6 and vasoactive agent like endothelin-1 (ET-1) promoting PASMC proliferation. Krüppel-like factor 5 (KLF5), is a zinc-finger-type transcription factor implicated in the regulation of cell differentiation, proliferation, migration and apoptosis. Recent studies have demonstrated the implication of KLF5 in tissue remodeling in cardiovascular diseases, such as atherosclerosis, restenosis, and cardiac hypertrophy. Nonetheless, the implication of KLF5 in pulmonary arterial hypertension (PAH) remains unknown. We hypothesized that KLF5 up-regulation in PAH triggers PASMC proliferation and resistance to apoptosis.

**Methods and results**

We showed that KFL5 is upregulated in both human lung biopsies and cultured human PASMC isolated from distal pulmonary arteries from PAH patients compared to controls. Using stimulation experiments, we demonstrated that PDGF, ET-1 and IL-6 trigger KLF-5 activation in control PASMC to a level similar to the one seen in PAH-PASMC. Inhibition of the STAT3 pathway abrogates KLF5 activation in PAH-PASMC. Once activated, KLF5 promotes cyclin B1 upregulation and promotes PASMC proliferation and triggers survivin expression hyperpolarizing mitochondria membrane potential decreasing PASMC ability to undergo apoptosis.

**Conclusion**

We demonstrated for the first time that KLF5 is activated in human PAH and implicated in the pro-proliferative and anti-apoptotic phenotype that characterize PAH-PASMC. We believe that our findings will open new avenues of investigation on the role of KLF5 in PAH and might lead to the identification of new therapeutic targets.

## Background

Pulmonary arterial hypertension (PAH) is a vascular disease that is mainly restricted to small pulmonary arteries. PAH occurs in rare idiopathic and familial forms, but is more commonly part of syndromes associated with connective tissue diseases, anorexigen use, HIV or congenital heart disease. This syndrome of obstructed, constricted small pulmonary arteries (PA) has been attributed to abnormalities in the blood content of some neurotransmitters and cytokines, namely increases in serotonin, IL-6, PDGF and endothelin-1 [1-4]. We recently demonstrated that the increase in these circulating vasoactive agents triggers in pulmonary artery smooth muscle cells (PASMC) the activation of the nuclear factor of activated T-cells (NFAT) contributing to increase [Ca^2+^]_i_-mediated PASMC proliferation [5,6]. Moreover, we showed a sustained increase in the oncoprotein survivin, decreasing mitochondrial-dependent apoptosis [7]. The fact that the PAH phenotype is preserved in cultured PASMC isolated from PAH patients suggests that the PAH phenotype is sustained independently of the circulating growth factors or agonists but requires genetic remodeling processes [8,9]. Moreover, despite recent therapeutic advances such as endothelin-1 receptor blockers (e.g. bosentan) [10], type 5 phosphodiesterase inhibitors (e.g. sildenafil) [11] or PDGF receptor blockers (e.g. imanitib) [12], mortality rates remain high [13].

Krüppel-like factor 5 is a zinc finger transcription factor that belongs to a family known as the Sp/KLF factors, and is implicated in important biological functions including cell proliferation, apoptosis, development, and oncogenic processes [14-16]. In Vascular Smooth Muscle Cells (VSMC) KLF5 regulates expression of the embryonic form of smooth muscle myosin heavy chain (SMemb/NMHC-B), which is selectively expressed in the proliferative dedifferentiated smooth muscle phenotype. In systemic vessels, KLF5 is expressed in proliferating smooth muscle cells of coronary artery lesions [17], and expression of this factor in lesions is clinically associated with restenosis and cardiac allograft vasculopathy [17]. KLF5 expression is therefore associated with proliferating smooth muscle cells in the cardiovasculature [18,19]. However, it had yet to be shown whether KLF5 is activated in PAH-PASMC and whether it's implicated in PASMC proliferation and apoptosis.

## Materials and methods

All experiments were performed in accordance to the Université Laval's Ethic and Biosafety Committee (protocol number 20142) and the Centre Hospitalier Universitaire de Québec's Ethic's Committee.

### Human tissue samples

All patients gave written informed consent before the study. Healthy lung tissues (controls) were obtained during lung resection for tumors. Only the healthy parts of the lungs were used in this study. All the PAH tissues were from lung explants from transplant or autopsy (Table 1).

**Table 1:** Patients providing tissue

	Patient type	Sex	Age	Mean PA pressure (mmHg)	Medications	PVR (dyne*sec)/cm^**5**^	Lung tissue
1	Control (Benign tumor)	F	35	ND	None	ND	Yes

2	Control (Lung Cancer)	F	38	ND	None	ND	Yes

3	Control (Benign tumor)	M	45	ND	None	ND	Yes

4	Control (Lung Cancer)	M	51	ND	None	ND	Yes

5	Control (Hodgkin)	M	48	ND	None	ND	Yes

6	Control (Benign tumor)	F	44	ND	None	ND	Yes

7	Control (Lung Cancer)	F	47	ND	None	ND	Yes

8	Control (Lung Cancer)	F	50	ND	None	ND	Yes

9	iPAH	F	58	56	Epoprostenol/Lasix/Coumadin	1709	Yes

10	iPAH	F	36	67	Epoprostenol/Lasix/Coumadin	2274	Yes

11	SSC-PAH	F	55	48	Epoprostenol/Lasix/Coumadin	980	Yes

12	PAH group1	F	64	59	Bosentan/Lasix	926	Yes

13	PAH group1	M	72	39	Lasix/Sitaxsentan	550	Yes

14	PAH group1	M	58	42	Epoprostenol/Lasix/Coumadin	991	Yes

15	PAH group1	F	51	51	Lasix/coumadin	1199	Yes

16	PAH group1	F	48	73	Epoprostenol/Lasix	1800	Yes

17	PAH group1	F	51	41	Lasix	990	Yes

18	PAH group1	F	68	37	Lasix/coumadin/Norvasc	544	Yes

### Cell culture

Cells were used in the first to sixth passage. Pulmonary arterial endothelial cell (PAEC), isolated form healthy human pulmonary arteries, were bought form the Cell application inc. (#302K-05a). PAH-PASMC were obtained from ≈1, 5 μm-diameter small pulmonary arteries from 2 males with iPAH (31 and 48 years old) and 1 female with PAH group 1 (54 years old) from lung explants. Age and sex matched control PASMC (3 males 45; 21; 64 years old and 2 females 17 and 35 years old)) were used. All patients had right catheterization that confirmed pulmonary hypertension (mean PAP greater than 25 mmHg at rest). PASMC were grown in high-glucose DMEM supplemented with 10% Fetal Bovine Serum (Gibco, Invitrogen, Burlington, ON, Canada) and 1% antibiotic/antimytotic (Gibco, Invitrogen, Burlington, ON, Canada) [20]. STAT3 was inhibited by a specific siRNA 20 nM for 48 h (Applied Biosystems Canada) and KLF5 was inhibited by a specific siRNA (30 nM for 48 h; Applied Biosystems Canada). Control PASMC were exposed to either PDGF (30 nG/mL); endothelin-1 (10 nM) or IL6 (20 ng/mL) (all from EMB Canada). All experiments were realized with a proper control siRNA scramble (siScr).

### In vivo model rats

Male Sprague-Dawley rats were used in this study. Monocrotaline (Sigma) was injected s.c. (60 mg/kg) to induce PAH. Once PAH is established (2 weeks post MCT injection), rats were treated by nebulization directly in the lungs of KLF5 siRNA (20 μM). PA pressure measurements have been realized by right heart catheterization to evaluate PAH severity.

### Quantitative RT-PCR

To measure KLF5 expression (using assay from Applied Biosystem), total mRNA was extracted from PAH-PASMC or control PASMC using trizol protocol. Quantification of mRNA expression was performed as previously described [6].

### Western Blot

Total protein extraction was realized on PASMC. KLF5 (Millipore; 1/500), PY705-STAT3 (Cell signaling; 1/1000), STAT3 (Cell signaling; 1/1000), Survivin (Cell signaling; 1/1000) and Cyclin B1 (Sigma Aldrich; 1/1000) were quantified and normalized to the smooth muscle actin (Santa cruz; 1/400) as previously described [6]. Evaluation for PY705-STAT3/STAT3 and KLF5/SM-actin were obtained from the same gel after stripping (30 min at 50 degrees).

### Confocal microscopy

TMRM, TUNEL, PCNA, Ki67 and AnnexinV were measured as previously described [6,21].

### Statistical Analysis

Values are expressed as fold change or mean ± SEM. Unpaired Student's *t *tests were used for comparisons between two means. For comparisons between more than two means we used one-way ANOVA followed by a Dunn's test. A *P *value < 0.05 was considered statistically significant (and indicated with asterisks *).

## Results

### KLF5 is upregulated in human and rodent pulmonary hypertension

**Figure 1:**
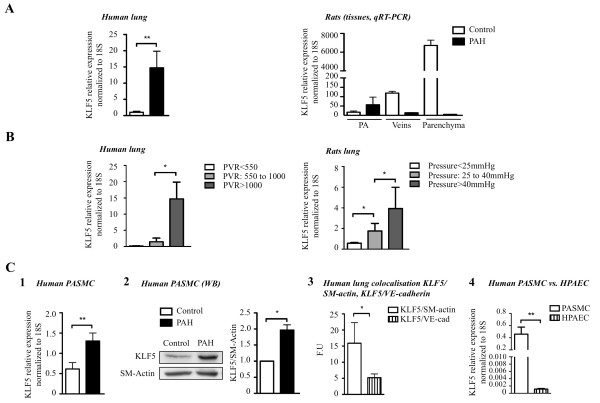
**KLF5 expression is increased in PAH**. **A) **KLF5 expression is measured by qRT-PCR in human lungs from patients with or without PAH. KLF5 is increased in PAH-patient (n = 10) compared to control patients (n = 8). KLF5 expression is also increased in distal pulmonary arteries (PA) of MCT-PAH-rats versus control rats (n = 10). **B) **KLF5 upregulation (measured by qRT-PCR) is correlated with PAH severity in human lung evaluated by pulmonary vascular resistance (PVR). Similarly, KLF5 expression in rat lungs is correlated with mean PA pressure upregulation. **C) **KLF5 upregulation (measured by (**1**) qRT-PCR or (**2**) Western blot) is confirmed in human pulmonary artery smooth muscle cell (PASMC) isolated from healthy patients or PAH patients (**3&4**). All qRT-PCR results were normalized to 18S gene expression and protein expression was measured by Western blot were normalized to SM-actin (*, P < 0.05; **, P < 0.01).

To investigate the expression pattern of KLF5 in normal and pulmonary hypertensive lungs, we examined KLF5 expression levels in 1) lung biopsies from 10 individuals with non-familial PAH compared to biopsies from 8 individuals without pulmonary hypertension, 2) lungs from 5 rats with monocrotaline-induced pulmonary hypertension compared to 10 control littermates (Figure 1A). We found increased levels of KLF5 in human and rodent pulmonary hypertensive lung tissues compared to normotensive lung samples. Within the lungs, KLF5 upregulation in PAH is confined to the distal PA (< 400 μm) (Figure 1A).

To test whether KLF5 upregulation is correlated with disease progression, we studied humans and rats with varying degrees of PAH. In both human subjects and rodents, KLF5 levels in the lung correlated directly with the severity of PAH, as measured by pulmonary vascular resistance (PVR) in humans and mean PA pressure in rodents (Figure 1B). Our results indicate that levels of KLF5 correlate with the severity of PAH in humans and experimental pulmonary hypertension.

To determine whether KLF5 expression in the pulmonary circulation is restricted to PASMC we measured KLF5 mRNA in human PASMC and PAEC. As shown in Figure 1C, KLF5 is preferentially expressed in PASMC. This finding was confirmed by co-localization studies between KLF5 and smooth muscle actin (marker of smooth muscle cell) or VE cadherin (marker of endothelial cells) in human lung biopsies. As shown, a greater co-localization was found between KLF5 and smooth muscle actin than KLF5 and VE cadherin. This finding confirms our qRT-PCR data and thus, that KLF5 is significantly more expressed in PASMC than PAEC (Figure 1C). Hence, for the rest of the study we have focused our research on PASMC. To determine whether KLF5 is upregulated in PAH-PASMC we used human cultured PASMC in the first to third passage. PAH-PASMC were obtained from ≈1, 500 μm-diameter small pulmonary arteries from 2 males with iPAH (31 and 48 years old) and 1 female with PAH group 1 (lupus; 54 years old) from lung explants [7]. All patients had right catheterization that confirmed pulmonary hypertension (mean PAP greater than 25 mmHg at rest). Age and sex matched control PASMC (3 males 45; 21; 64 years old and 2 females 17 and 35 years old)) were used. KLF5 upregulation between control and PAH was confirmed by qRT-PCR and Western blot (WB) (Figure 1C). Note that no significant differences in KLF5 expression was found among the control patients and among the PAH patients (**not shown**). Therefore for the rest of the study all 5 control-PASMC and the 3 PAH-PASMC cell lines were used for every cell-based experiment.

### KLF5 is activated in PAH-PASMC through a STAT3 dependent mechanism

**Figure 2:**
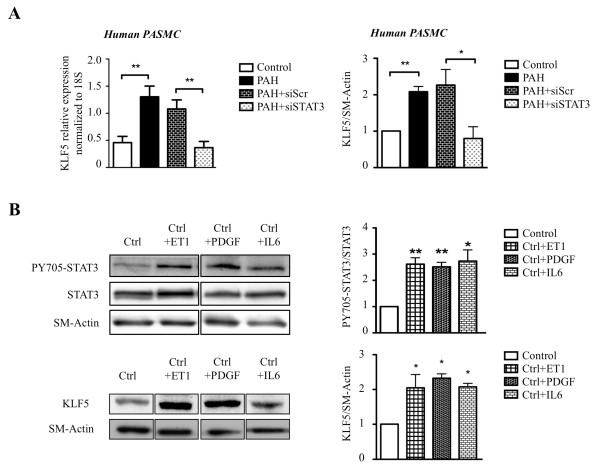
**STAT3 activation increases KLF5 expression**. **A) **STAT3 inhibition by siRNA decreases KLF5 RNA expression measured by qRT-PCR in human PAH-PASMC and protein expressions were measured by Western blot. **B) **STAT3 activation (i.e. PY705-STAT3/STAT3 ratio) and KLF5 expression were measured in control cells with or without stimulation (ET-1, PDGF, IL-6), by Western blot. Our results show that ET1, PDGF and IL6 stimulations induce STAT3 activation and upregulation of KLF5 expression. qRT-PCR results were normalized to 18S gene expression and protein expression measured by Western blot were normalized to SM-actin (*, P < 0.05; **, P < 0.01).

The Signal Transducers and Activators of Transcription (STATs) family is composed of 7 isoforms (STAT1; 2; 3; 4; 5A; 5B and 6) with STAT3 being the most important one in cardiovascular diseases [22,23]. STATs are activated (i.e. phosphorylated) by either the Janus-activated kinase (JAKs) or the Src family kinases in response to cytokines (like IL-6, TNF...) [24], growth factors (like PDGF...) [24] or agonists (like ET1, AngII...) [25], all of which are implicated in PAH [26-28]. Interestingly, a recent study in stem cells revealed a putative regulation of KLF5 by STAT3 [29]. To demonstrate the implication of STAT3 in the regulation of KLF5 expression in PAH, KLF5 expression was measured in PAH-PASMC and control PASMC in the presence or absence of STAT3 siRNA. Compared to siScr treated PAH-PASMC, siSTAT3 treated PAH-PASMC had significantly reduced KLF5 expression (Figure 2A). These findings suggest that STAT3 activation accounts for KLF5 expression in PAH-PASMC. To further confirm the implication of STAT3/KLF5 axis in PAH, healthy-PASMC were treated for 48 h with increasing doses of pro-PAH factors [26-28] like ET1 (10 nM), PDGF (30 ng.mL^-1^) and IL-6 (20 ng.mL^-1^). As expected, pro-PAH factors, increased STAT3 activation (PY705-STAT3/STAT3 ratio) in ET1, PDGF and IL-6 treated PASMC respectively (Figure 2B). Interestingly, we found that in healthy-PASMC treated with pro-PAH factors (ET1, PDGF or IL-6) the activation of STAT3 promotes KLF5 expression (Figure 2B).

### KLF5 inhibition decreases PAH-PASMC proliferation and resistance to apoptosis

**Figure 3:**
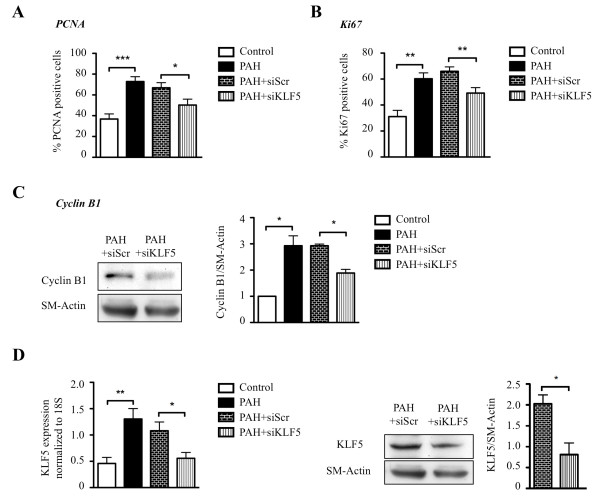
**KLF5 inhibition decreases proliferation**. **A&B) **PASMC cell proliferation was evaluated by PCNA and Ki67 immunostaining (nuclear translocation). PAH PASMC are significantly more proliferative than control PASMC and KLF5 inhibition (siRNA) decreases PAH-PASMC proliferation compared to siScr. **C) **Cyclin B1 (a proliferation biomarker) is increased in PAH-PASMC and KLF5 inhibition decreases significantly cyclin B1 expression. **D) **KLF5 inhibition in PAH-PASMC has been evaluated by qRT-PCR and Western blot. KLF5 inhibition, in PAH-PASMC using siRNA, decreases significantly KLF5 expression compared to the appropriate control: PAH-PASMC with siRNA Scramble (siScr). qRT-PCR were normalized to 18S gene and Western blot were normalized to SM-Actin (*, P < 0.05; **, P < 0.01; ***, P < 0.001).

**Figure 4:**
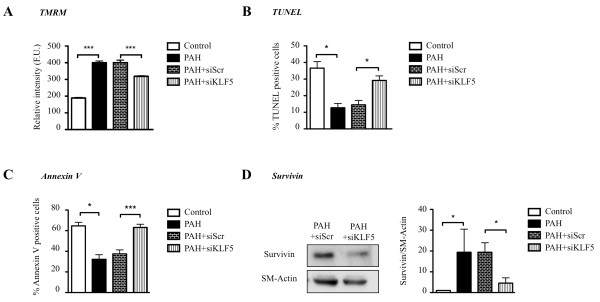
**KLF5 inhibition promotes apoptosis**. **A) **Mitochondrial membrane potential was measured by TMRM in confocal microscopy. PAH- PASMC are more hyperpolarized than control (more red) and KLF5 inhibition (siRNA) depolarized PAH-PASMC. **B&C) **Apoptosis was also evaluated by TUNEL and Annexin V staining in PASMC. PAH-PASMC are more resistant to starvation induced apoptosis (0, 1%FBS) than control PASMC. KLF5 inhibition in PAH-PASMC promotes apoptosis. **D) **Survivin expression (measured by Western blot) is increased in PAH-PASMC compared to control cells and KLF5 inhibition (siRNA) decreased survivin expression. Western blot were normalized to SM-actin (*, P < 0.05; ***, P < 0.001).

To study the effect of KLF5 on PASMC proliferation and apoptosis *in vitro*, cultured human PAH-PASMC were either exposed to 10% FBS to promote proliferation or 0.1% FBS to promote apoptosis [6]. When compared to control PASMC containing a low level of KLF5, PAH-PASMC displayed higher cell proliferation rate and resistance to induced apoptosis (Figure 3&4).

Using Ki67 and PCNA, we measured the effect of KLF5 inhibition on PAH-PASMC proliferation. The KLF5 inhibition using siRNA in PAH-PASMC decreases proliferation to the level seen in control-PASMC (Figure 3A&3B). Note that siScr has no effect. The increased proliferation seen in PAH-PASMC is associated with a KLF-5-dependent up-regulation of cyclin B1, confirming previous findings in systemic vessels [30] and cancer [31] (Figure 3C). Moreover, we confirmed that siKLF5 decreased significantly KLF5 (Figure 3D).

Resistance to apoptosis observed in PAH-PASMC has been linked to mitochondrial membrane potential (ΔΨ_m_) hyperpolarization, which would block the release of pro-apoptotic mediators like cytochrome *c *[6,20,21]. Using tetramethylrhodamine methyl ester (TMRM), we measured whether KLF5 inhibition can affect mitochondrial hyperpolarization. We observed that KLF5 inhibition in PAH-PASMC depolarizes ΔΨ_m _to a level similar to that observed in control-PASMC (Figure 4A). Finally, mitochondrial depolarization induced by KLF5 inhibition in PAH-PASMC increases serum starvation-induced apoptosis (TUNEL, annexin V) (Figure 4B&4C). We previously published that ΔΨ_m _hyperpolarization was associated to the upregulation of the oncoprotein survivin and that survivin inhibition in PAH-PASMC reverses mitochondrial hyperpolarization, promoting apoptosis. Nonetheless, the mechanism accounting for survivin expression in PAH-PASMC remains elusive. Recently, in cancer, KLF5 has been shown to promote survivin expression. To determine whether the survivin upregulation in PAH-PASMC is KLF5 dependent, survivin expression was measured in PAH-PASMC in presence and absence of KLF5 siRNA. We found that KLF5 inhibition decreases survivin expression (Figure 4D) and thus provides a putative mechanism accounting for the KLF5-dependent ΔΨ_m _hyperpolarization seen in PAH-PASMC.

### KLF5 inhibition *in vivo *improves PAH

**Figure 5:**
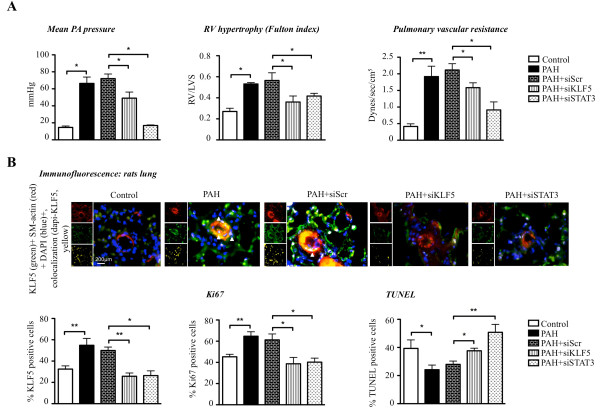
***In vivo*, KLF5 inhibition reverses PAH development**. **A) **Mean PA pressure was measured on closed chest rats by right catheterization. The mean PA pressure is increased significantly in PAH, whereas KLF5 and STAT3 inhibition (siRNA) decrease the pulmonary pressure. The right ventricular hypertrophy, evaluated by Fulton index measurement, is reversed with KLF5 and STAT3 inhibition. Pulmonary Vascular Resistances (PVR) are increased in PAH rats and KLF5 or STAT3 inhibition decreased them. **B) **KLF5 activation has been evaluated in distal pulmonary arteries from rats by immunofluorescence. Nuclear colocalization of KLF5 is increased in PAH-PASMC from distal PA compared to healthy PA and KLF5 and STAT3 inhibition decrease KLF5 translocation. Proliferation and apoptosis have been evaluated by immunofluorescence (respectively by Ki67 and TUNEL). Proliferation increases in PAH-PA compared to control PA was decreased after treatment with siKLF5 or siSTAT3. Apoptosis resistance of the PA-PAH is reversed after KLF5 and STAT3 inhibition (*, P < 0.05; **, P < 0.01; ***, P < 0.001).

To confirm the STAT3-dependent regulation of KLF5, *in vivo *rats were injected with monocrotaline for 4 weeks. Once PAH established (increased PA pressure and increased right ventricle wall hypertrophy (RVH)), rats were nebulized with either siSTAT3 or siScr for 2 weeks. Effects on PAH (mean PA pressure; RVH) were measured by right catheterization and Fulton index. KLF5 expression was measured immunofluorescence in distal PA (Figure 5B). As shown, siSTAT3 decreases KLF5 expression and PAH in distal PA, confirming our *in vitro *findings (Figure 2A). Note that the efficiency of our therapeutic intervention was confirmed *i.e*. siSTAT3 significantly decreases STAT3 in distal PA. Among all the rats treated with siSTAT3, the rats showing the greater KLF5 downregulation had the lower mean PA pressure, suggesting that KLF5 is implicated in the etiology of PAH. To determine whether KLF5 has direct therapeutic potential for PAH, monocrotaline rats (with established PAH 2 weeks post injection) were nebulized with either siKLF5 or siScr for 2 week. Efficiency of our intervention was confirmed in distal PA where KLF5 expression is significantly decreased (Figure 5B). As shown, KLF5 inhibition improves PAH by reducing RVH and mean PA pressure (Figure 5A).

## Discussion

Here we show that KLF5 is etiologically associated with the development of PAH, and believe that we have opened a new avenue for PAH treatment. KLF5 is expressed in established human and experimental PAH. Its expression is dependent of the activation of STAT3. We provided evidences that KLF5 inhibition improves PAH by 1) the inhibition of cyclin B1 and PASMC proliferation and 2) the depolarization of PASMC mitochondria by inhibiting survivin activation thus increasing apoptosis. These findings not only confirm what has been shown in cancer [16,31,32] and systemic vascular smooth muscle cells [33,34] in which KLF5 inhibition decreases proliferation through cell cycle protein inhibition like cyclin B1 and p21 upregulation, but also provide a better demonstration of the involvement of KLF5 in mitochondrial-dependent apoptosis. In fact, we provide for the first time evidences that KLF5 is implicated in mitochondrial membrane potential regulation through the upregulation of the oncoprotein survivin. We have previously extensively demonstrated the mechanism of mitochondrial membrane potential regulation by survivin in PAH-PASMC [7], but had not elucidated the mechanism accounting for its upregulation. Our new findings propose that KLF5 activation might explain such upregulation. In fact, we showed that KLF5 inhibition decreases survivin expression in PAH-PASMC. This is associated with a significant mitochondrial depolarization. This finding could be of great therapeutic interest as it provides a new insight on the regulation of survivin that is implicated in many cancer and cardiovascular diseases [35-39]. Thus, our findings might not be limited to PAH but can be extend to many other diseases including cancer. To this end, a recent report has demonstrated a link between KLF5 and survivin in cancer [40].

Previous studies reported that KLF5 is implicated in embryonic stem cells and VSMC differentiation contributing to vascular lesions [34,41]. This aspect might be implicated in the vascular lesions seen in PAH patients. Indeed, vascular lesions such as remodeled arteries and plexiform lesions are seen in patients with PAH. Studies have demonstrated the implications of abnormal stem cells in this phenomenon [42]. KLF5 implication in such processes cannot be ruled out.

The activation of KLF5 axis that we described likely has a multifactorial etiology in PAH. However, KLF5 might be a critical integrator of multiple signaling pathways and its downstream effects might explain several and important features of PAH. *In vivo*, endothelial dysfunction is recognized as one of the earliest abnormalities in PAH, resulting in a well-recognized imbalance of endothelium-derived vasoactive factors; with increased vasoconstrictors (like endothelin [28], thromboxane [1] and decreased vasodilators (like NO or prostacyclin [1]). Recently, KLF5 has been shown to be implicated in endothelial dysfunction [43]. In addition, increased circulating growth factor (like PDGF) [27] and cytokines (like IL-6, MCP-1...) [26] have been reported in PAH, and there is numerous evidences demonstrating the implication of KLF5 in both growth factor and cytokines production regulation [18,43-45].

Finally, we have previously extensively demonstrated the implication of HIF-1 in triggering mitochondrial and [Ca^2+^]_i _dysfunction sustaining the pro-proliferative and anti-apoptotic phenotype seen in PAH-PASMC [5,46]. Interestingly, Mori *et al*. have shown that cooperation between HIF-1 and KLF5 might exist [47]. Indeed, KLF5 inhibition decreases the expression of several HIF-1-regulated genes in cancer cells, while HIF-1 inhibition affects KLF5 expression [47]. These findings in cancer cells implied that interaction between HIF-1/KLF5 in PAH-PASMC might exist. Thus, all these reports suggest that KLF5 might play a critical role in the etiology of PAH. Surprisingly no studies have been performed on the topic.

The mechanisms accounting for KLF5 upregulation in PAH remain to be established. Recently, a study from Cheng et al [48] has suggested the implication of the microRNA 145 (miR-145). In their study, they provided evidences that the downregulation of miR-145 promotes vascular neointimal lesion formation through the upregulation of KLF5. Nonetheless, downregulation of miR-145 in PAH, has not been demonstrated [49]. We believe that one of the initial events in PAH, regardless of the specific cause, is the activation of the miR-204/STAT3 axis [50], which increases KLF5 expression (Figure 2), supporting the notion that KLF5 induction is an early event during PAH. Thus, KLF5 upregulation can be caused by many diverse conditions that lead to PAH in addition to hypoxia, including growth factors, vasoactive molecules [50,51], or viral infection of PASMC [52]. The fact that STAT3 activation may occur only in pulmonary, not systemic, vasculature [50] explains the selective induction of KLF5 in the pulmonary circulation. This finding strengthens the argument that KLF5 therapeutic targeting may achieve relative selectivity for the pulmonary circulation in PAH. Although many more experiments are needed in order to determine whether this regulation is direct or indirect, this finding is of great interest. In fact, STAT3 has been associated with many feature of PAH including BMPR2 downregulation (hallmark of PAH) [53]. Involvement of KLF5 in the STAT3-mediated effects in PAH will certainly open new avenues of investigation. Finally, we showed that both indirect (through STAT3 inhibition) and direct KLF5 inhibition (nebulized siRNA) improve PAH. Nonetheless, we should note that STAT3 inhibition showed a greater efficiency than siKLF5. This is not surprising as STAT3 has been implicated in many features characterizing PAH, including miRNA such as miR-204, Pim1; NFAT; BMPR2 [6,50,51,53]

## Conclusion

Although our findings will need to be repeated in greater amount of patients and at different stages of PAH to have a better understanding of the role of KLF5 in PAH, nonetheless our study suggests for the first time the implication of KLF5 in the etiology of human PAH. Moreover we provide evidences that its activation might account for many feature seen in PAH, including PASMC proliferation, mitochondrial hyperpolarization, survivin expression and resistance to apoptosis. Because, pulmonary arterial hypertension is a rapidly lethal disease for which treatments are limited, we believe that our findings will open the door to new avenues of investigation and potentially future therapies for PAH.

## Competing interests

The authors declare that they have no competing interests.

## Authors' contributions

AC contributed in all the data experiments, analysis and elaborated the figures. VLT performed the Western blot, their analysis and contributed to immunofluorescence experiments. MB, JM and MHJ contributed in qRT-PCR analysis and amelioration of the manuscript. MC, MB, RP and CL contributed in analyzed, *in vivo *measurements and cell culture experiments. SP helps in manuscript criticism and collaborates for human tissue experiments. SB designed the study, supervised the overall study and wrote the manuscript. All authors have read and approved the manuscript.
